# Subtle Differences in Symbiont Cell Surface Glycan Profiles Do Not Explain Species-Specific Colonization Rates in a Model Cnidarian-Algal Symbiosis

**DOI:** 10.3389/fmicb.2018.00842

**Published:** 2018-05-01

**Authors:** John E. Parkinson, Trevor R. Tivey, Paige E. Mandelare, Donovon A. Adpressa, Sandra Loesgen, Virginia M. Weis

**Affiliations:** ^1^Department of Integrative Biology, Oregon State University, Corvallis, OR, United States; ^2^Department of Chemistry, Oregon State University, Corvallis, OR, United States

**Keywords:** Aiptasia, *Exaiptasia pallida*, glycans, lectin array, recognition, specificity, *Symbiodinium*

## Abstract

Mutualisms between cnidarian hosts and dinoflagellate endosymbionts are foundational to coral reef ecosystems. These symbioses are often re-established every generation with high specificity, but gaps remain in our understanding of the cellular mechanisms that control symbiont recognition and uptake dynamics. Here, we tested whether differences in glycan profiles among different symbiont species account for the different rates at which they initially colonize aposymbiotic polyps of the model sea anemone Aiptasia (*Exaiptasia pallida*). First, we used a lectin array to characterize the glycan profiles of colonizing *Symbiodinium minutum* (ITS2 type B1) and noncolonizing *Symbiodinium pilosum* (ITS2 type A2), finding subtle differences in the binding of lectins *Euonymus europaeus* lectin (EEL) and *Urtica dioica* agglutinin lectin (UDA) that distinguish between high-mannoside and hybrid-type protein linked glycans. Next, we enzymatically cleaved glycans from the surfaces of *S. minutum* cultures and followed their recovery using flow cytometry, establishing a 48–72 h glycan turnover rate for this species. Finally, we exposed aposymbiotic host polyps to cultured *S. minutum* cells masked by EEL or UDA lectins for 48 h, then measured cell densities the following day. We found no effect of glycan masking on symbiont density, providing further support to the hypothesis that glycan-lectin interactions are more important for post-phagocytic persistence of specific symbionts than they are for initial uptake. We also identified several methodological and biological factors that may limit the utility of studying glycan masking in the Aiptasia system.

## Introduction

Cnidarians are basal marine metazoans such as corals and anemones, many of which can associate with dinoflagellate endosymbionts belonging to the genus *Symbiodinium* (Trench, 1993). These mutualisms form the foundation of coral reefs, one of the most biodiverse and economically important marine ecosystems (Moberg and Folke, 1999). Though usually beneficial, the partnership can be a weak point for corals because it makes them susceptible to light and temperature stress (Weis, 2008), which drive the potentially lethal dissociation known as “coral bleaching.” As a result, reefs are threatened globally by climate change (Hoegh-Guldberg, 1999), most dramatically exemplified by the recent, multi-year bleaching event which affected the majority of the Great Barrier Reef (Hughes et al., 2017). As corals decline, renewed effort has been placed on characterizing the cell biology of the cnidarian-*Symbiodinium* association (Weis et al., 2008; Davy et al., 2012). In particular, it is critical to describe symbiosis establishment and persistence under normal conditions, in order to better understand exactly how dysbiosis develops under stress.

The genus *Symbiodinium* can be divided into several major divergent lineages (“clades”), each of which is composed of many reproductively isolated species (“types”), each of which in turn is composed of numerous individuals (“strains”) (for review, see Thornhill et al., 2017). At each level of organization, there is tremendous physiological diversity (Suggett et al., 2017). For example: within the genus, members of Clade D are more likely to be stress tolerant than members of other clades (LaJeunesse et al., 2014); within Clade B, *S. minutum* is warm-water adapted while *S. psygmophilum* is cold-water adapted (LaJeunesse et al., 2012); and within *S. psygmophilum*, some strains grow faster than others and have unique gene expression profiles (Parkinson and Baums, 2014; Parkinson et al., 2016). These dramatic functional differences help explain physiological variability among corals that associate with different symbiont species (Sampayo et al., 2008), the tight correspondence between the evolutionary history of certain hosts and symbionts (Thornhill et al., 2014), and why symbionts cannot be easily switched without incurring costs (Matthews et al., 2017).

Given this functional diversity, it is critical for many coral hosts to maintain highly specific symbiont associations. And yet, most corals must re-establish their symbiotic partnerships anew each generation – a paradoxical arrangement that may have evolved to increase survival during early larval stages (Hartmann et al., 2017). Therefore, mechanisms must be in place for hosts to acquire potential symbionts, reject incorrect partners, and accept correct ones; at the same time, symbionts must be able to find appropriate hosts (Davy et al., 2012). In other symbiotic systems, the ability to recognize and discern potential partners often involves signaling between cell surface molecules on both organisms: symbiont glycans (mono- or polysaccharides, often conjugated to proteins or lipids) and host lectins (proteins that bind to glycans). Recent genomic work has revealed that symbiotic *Symbiodinium* and their cnidarian hosts are enriched for genes and proteins associated with glycan and lectin pathways, and these targets are under positive selection (Baumgarten et al., 2015; Neubauer et al., 2017; Liu et al., unpublished).

Most relevant in the context of symbiosis establishment, cell surface glycan-lectin interactions may regulate initial recognition events when host and symbiont cells are first brought into contact (Trench, 1988; Markell et al., 1992). In a screening of various *Symbiodinium* spanning several major clades, Logan et al. (2010) identified unique glycan profiles for each species, potentially providing a foundation for hosts to distinguish different symbionts. In addition, different *Symbiodinium* species (Wolfowicz et al., 2016) and strains (Hawkins et al., 2016) colonize hosts at different rates, raising questions about to what extent these dynamics are mediated by glycan-lectin recognition events. Several previous efforts to alter symbiont colonization rates through cell surface glycan manipulation succeeded in reducing symbiont acquisition in aposymbiotic cnidarian hosts. Masking glycans with exogenous lectins has reduced colonization success in coral larvae several times (Wood-Charlson et al., 2006; Bay et al., 2011; Kuniya et al., 2015) and in experimentally bleached sea anemone polyps once (Lin et al., 2000). These studies also found that cleaving symbiont glycans with enzymes reduced colonization. Notably, glycan masking and enzymatic treatment does not affect all symbiont species equally, and does not always reduce colonization (Bay et al., 2011). These results mirror equivocal findings in the *Hydra-Chlorella* association (Jolley and Smith, 1980; Meints and Pardy, 1980), where it was concluded that glycan-lectin interactions do not play a role in primary recognition.

Although evidence for nonspecific symbiont uptake has also been provided for cnidarian-*Symbiodinium* associations (Colley and Trench, 1983), this work was performed before molecular techniques were available to distinguish different symbiont species. The only other glycan masking colonization experiment to include multiple *Symbiodinium* (that of Bay et al., 2011) used freshly isolated cells that potentially could have been contaminated with tissues from different hosts, which can alter uptake dynamics (Trench et al., 1981). Building on these efforts, we used cultured cells free of host contamination from two ecologically and evolutionarily divergent *Symbiodinium*, along with recently developed lectin array technology, to determine whether species-specific glycan profiles affected symbiont colonization rates in the model cnidarian sea anemone Aiptasia (*Exaiptasia pallida*).

## Materials and Methods

All raw data and R code for the following analyses can be found in the **Supplementary Data Sheet S1**. More detailed protocols can be accessed on protocols.io at dx.doi.org/10.17504/protocols.io.j8ucrww.

### Animal and Algal Maintenance

Animal host Aiptasia (*E. pallida*) polyps were experimentally bleached with menthol following Matthews et al. (2016) to render them aposymbiotic. The polyps were periodically checked under a fluorescent microscope to confirm the absence of symbiont cells. They were maintained at RT in darkness and fed *Artemia* nauplii three times every week. The female clonal line H2 was originally isolated from Hawaii and is a member of the globally distributed Aiptasia population (rather than the Florida lineage; see Thornhill et al., 2013). The GME line’s sex is unknown, but it was also isolated from Hawaii and belongs to the global genetic network.

Micro-algal symbiont *Symbiodinium* spp. cultures were grown in F/2 media in an incubator maintained at 26°C with a 12:12 L:D photoperiod and light intensity of 50 μmol quanta⋅m^-2^⋅s^-1^. *S. minutum* (ITS2 type B1), the homologous symbiont of Aiptasia (LaJeunesse et al., 2012; Thornhill et al., 2013), was represented by two cultures: Mf1.05b originally isolated as a contaminant from the surface of *Orbicella* (formerly *Montastrea*) *faveolata* from Florida, and FLAp2 originally isolated from Aiptasia from Florida. *S. pilosum* (ITS2 type A2), a nonsymbiotic species (Trench and Blank, 1987; LaJeunesse and Trench, 2000), was represented by strain Zs (also known as rt185 or CCMP 2461) originally isolated as a contaminant from *Zoanthus sociatus* from Jamaica.

### Lectin Array

To prepare samples for the lectin array, algal cultures were trypsinized following a modified protocol (Bay et al., 2011). For each strain of interest (Mf1.05b for *S. minutum* and Zs for *S. pilosum*), 1 mL of cultured algae (1 × 10^6^ cells⋅mL^-1^) was centrifuged at 1500 RCF for 15 min and resuspended in 2 mL 2X PBS. The rinse was repeated twice, then the final volume was divided into four replicate tubes (0.5 mL⋅tube^-1^). To each tube, 0.5 mL of 1 mM HCl and 2.48 μL of a 2.48 mg mL^-1^ trypsin stock were added. The tubes were incubated at RT in the dark for 2 h with gentle mixing every 20 min. They were then centrifuged at 15000 RCF to pellet algal debris. The supernatant was transferred to a new tube and shipped to RayBiotech, where the samples were run on Lectin Array 40 (GA-Lectin-40-16), which contains a suite of 40 lectins (Dan et al., 2016). The single array slide accommodated four replicates of each culture. Two replicates with obvious and uncorrectable artifacts were dropped from the analysis, leaving three technical replicates per culture.

### Glycan Recovery Rates

To prepare samples for establishing glycan recovery rates, N-linked glycoproteins (high mannose, hybrid, and complex oligosaccharides) were enzymatically cleaved between the *N-*acetylglucosamine (GlcNAc) moiety and the asparagine residues of the proteins in the cultures of interest (Mf1.05b for *S. minutum* and Zs for *S. pilosum*) using PNGase F (an amidase) following the protocol of Lin et al. (2000). For each replicate, 1 mL of cultured algae (0.5 × 10^6^ cells⋅mL^-1^) was incubated with PNGase F (0.1 μg⋅mL^-1^) or an equal volume of FSW as a control. After a 24 h incubation, algae were pelleted (500 RCF for 5 min) and resuspended in 1 mL filtered seawater (FSW). PNGase F exposures were staggered so that treatments represented 0 or 72 h of recovery after glycan cleavage.

Subsequently, algae were exposed to lectin Con A bound to fluorescent phycoerythrin at a final concentration of 5 μg⋅mL^-1^. Lectins were coupled using the Lightning-Link R-PE Antibody Labeling Kit (Novus Biologicals #703-0010) following the manufacturer’s protocol. There were three replicates for each combination of recovery time and exposure treatment. After a 2 h incubation, the algae were washed, pelleted (500 RCF for 5 min), and resuspended in 1 mL 3.3X PBS, then run on a CytoFLEX flow cytometer. *Symbiodinium* cell populations were identified via forward and side scatter and confirmed via chlorophyll autofluorescence (> 660 nm). Cells labeled with phycoerythrin were excited at 561 nm and captured in channel FL10 (585/42 BP). Detection gates were calibrated for phyocerythrin labeling using unstained algae of the same strain, and the percentage of stained cells per treatment was quantified from the total *Symbiodinium* cell population. Under control conditions, this experiment gave an estimate for Con A masking efficiency. Similar treatments were carried out to estimate *Euonymus europaeus* lectin (EEL)- and *Urtica dioica* agglutinin lectin (UDA)-masking efficiency.

### Glycan Masking and Colonization

Lectins determined to be differentially abundant between species on the array were used to mask algal cell surface oligosaccharide residues (**Supplementary Figure S1**). Cultured algae (3 × 10^6^ cells⋅mL^-1^) were incubated in 1 mL of a given lectin (100 μg⋅mL^-1^) or an equal volume of FSW as a control. Exposures lasted 2 h at RT with gentle mixing every 20 min. Prior to inoculation, algae were pelleted (500 RCF for 5 min) and resuspended in 3 mL FSW three times.

Inoculations were performed in 24-well flat-bottomed culture plates with anemones that had been starved for 24 h. Each treatment well contained a single aposymbiotic anemone with an oral disk diameter of ∼0.5 mm. To each well, 1 mL of FSW containing lectin-treated algae (1 × 10^6^ cells⋅mL^-1^) was added, along with 10 μL of brine shrimp homogenate to stimulate a feeding response. Positive controls were provided untreated algae, while negative controls were provided heat-killed algae or no algae (all negative controls lacked algae by the end of the experiments). Plates were then moved into an incubator maintained at 26°C with a 12:12 L:D photoperiod and light intensity of 15 μmol quanta⋅m^-2^⋅s^-1^. At 24 h post-inoculation, an additional 1 mL of FSW was added to each well to improve oxygenation and to resuspend algal cells. At 48 h post-inoculation, the polyps were transferred to a new plate with new FSW (leaving most algal cells behind). At 72 h post-inoculation, the water was replaced with 0.376 M MgCl_2_ in FSW to relax the polyps. They were then fixed in 4% paraformaldehyde in PBS for 1 h and stored overnight at 4°C in PBS.

Five experiments were performed to test the effect of various factors on symbiont colonization rates (for full details, see section “Results and Discussion”): (1) a test of unique glycan effects with Aiptasia (H2) and *S. minutum* (Mf1.05b) and glycan masking (lectins Con A, EEL, and UDA); (2) a test of feeding effects with Aiptasia (H2) and *S. minutum* (Mf1.05b) and glycan masking (lectin UDA) using anemones that had been starved for 120 h; (3) a test of symbiont strain effects with Aiptasia (H2) and *S. minutum* (Mf1.05b or FLAp2) and glycan masking (lectin Con A); (4) a test of host strain effects with Aiptasia (H2 or GME) and *S. minutum* (Mf1.05b) and glycan masking (lectin Con A); and (5) a test of cultured vs. freshly isolated symbionts (FIS) with Aiptasia (H2) and *S. minutum* (Mf1.05b) and glycan masking (lectin Con A). To generate FIS, symbiotic Aiptasia H2 anemones were homogenized in FSW and centrifuged at 500 RCF for 1 min. The supernatant containing host tissue was removed and the algal pellet was resuspended in FSW. This process was repeated three times, reducing but not eliminating host tissue contaminants.

### Visualization

To quantify colonization efficiency, anemones were observed under fluorescence microscopy. Given that colonization in adult Aiptasia has been shown to begin in the tentacles and oral disk with the column becoming colonized later (Gabay et al., 2018), fixed polyps were dissected to remove most of the column and prevent it from interfering with the area-based cell density measurement. The oral disk and tentacles were laid flat and mounted on a slide in a 90% glycerol in PBS mountant solution. Fluorescent images were captured on a Zeiss AxioObserver A1 microscope with an Axiovert ICm1 camera (Carl Zeiss AG, Jena, Germany) using the Cy3 (red) filter to observe algal cell chlorophyll autofluorescence and the GFP (green) filter to observe host green fluorescent protein. Oral disk area was excluded manually in ImageJ (Abramoff et al., 2004). Using the red channel images, algal cells within tentacles were automatically counted based on contrast using the ITCN ImageJ plugin. Using the green channel images, host tentacle surface area was determined based on contrast, also in ImageJ. Algal density was calculated as cell count divided by surface area.

### Statistics

Lectin arrays produce fluorescence values that are, in principle, identical to single-channel RNA microarray data, and can therefore be analyzed the same way. To determine which lectins were differentially abundant in the trypsinized lysates of the two algal species, the fluorescence data were imported into the R Statistical Environment and analyzed as a single-channel array experiment with the ‘*LIMMA*’ package (Smyth, 2005). The values were background corrected, normalized, and fit to linear mixed models. The top differentially abundant lectins were ranked by uncorrected *p*-values. For glycan recovery times, staining values were confirmed to be normally distributed and homoscedastic; they were analyzed via one-way ANOVA using the base ‘*stats*’ package in R. For the colonization experiments, differences in algal densities among treatments were also analyzed via one-way ANOVA or, in the case of any two-treatment comparison, a *t*-test. Negative control treatments were excluded, and the remaining values were log-transformed to achieve normality and homoscedasticity. Complementary Bayes factor analyses were performed using the ‘*BayesFactor*’ package.

## Results and Discussion

We investigated glycan-lectin interactions in the Aiptasia – *Symbiodinium* model cnidarian system. Taking a host-centric view for these experiments, we focused on the role of symbiont cell surface glycoproteins as microbe-associated molecular patterns by examining their effect on colonization rates – a phenotype likely mediated by both host and symbiont biology. We used experimentally bleached adult polyps because Aiptasia larvae remain difficult to generate and maintain. Given the dependence of each manipulation on the outcome of the previous trial, we combine the Section “Results and Discussion” for every analysis presented below.

### Species-Specific Glycan Profiles

**FIGURE 1:**
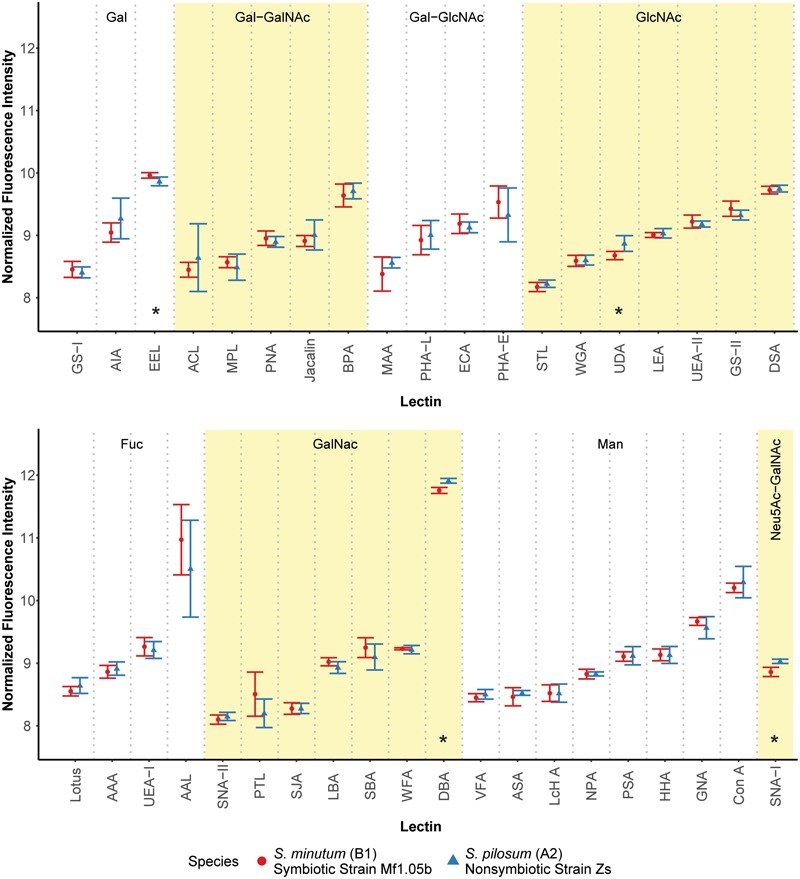
Fluorescence intensity of symbiont cell surface glycans bound to the lectin array. Lectins are organized by their carbohydrate specificity (indicated at the top of each graph), corresponding to alternately shaded regions. Lectins with asterisks were differentially abundant on the surface of symbiotic vs. nonsymbiotic *Symbiodinium* species (unadjusted *p* ≤ 0.10, *n* = 3 technical replicate arrays). Error bars represent SD. Fuc = L-Fucose, Gal = D-Galactose, GalNAc = *N*-Acetylgalactosamine, GlcNAc = *N*-Acetylglucosamine, Man = Mannose, Neu5Ac = *N*-Acetylneuraminic acid.

We first hypothesized that different algal species from different clades with different ecologies would exhibit unique glycan profiles. To test this, we used a commercially available 40-lectin array to characterize the binding of glycans cleaved from the cell surfaces of two *Symbiodinium* species: the homologous symbiont *S. minutum* (ITS2 type B1), which is readily taken up by aposymbiotic anemone polyps and other cnidarian larvae (Belda-Baillie et al., 2002; Wolfowicz et al., 2016), and the nonsymbiotic *S. pilosum* (ITS2 type A2), which is incapable of establishing symbioses with cnidarians (Markell et al., 1992; LaJeunesse, 2001; Wolfowicz et al., 2016). Lectin-based microarray technology has emerged as a useful analytical tool in glycobiology (Hsu and Mahal, 2009). It allows for rapid profiling of glycosylation present in various biological systems by using the well-defined specificity and affinity of known lectins (accessible from the Consortium of Function Glycomics^1^). It is also effective at detecting subtle variations in the quantitative levels of exposed carbohydrate residues, rather than just presence/absence (Pilobello et al., 2007). Overall, we found that glycan-lectin binding patterns were quite similar across the two species (**Figure 1** and **Supplementary Figure S2**), with only four lectins showing marginally differential binding at *p* ≤ 0.10 prior to adjustment for multiple comparisons, and no significant differences after correction (**Supplementary Table S1**).

These results stand in contrast to previous efforts using low resolution electrophoretic analysis to distinguish between cell surface molecule profiles (Markell et al., 1992) and high resolution flow cytometry to sort cells labeled with fluorescent lectin probes (Logan et al., 2010). In the flow cytometry study, the authors found large differences in fluorescence intensities among cultures of the same two species tagged with six of the same lectins. A possible explanation for this discrepancy is that we normalized the samples for the lectin array to an equal amount of cleaved oligosaccharide, and thus we measured the relative proportions of the glycans rather than estimating their absolute abundances. On average, *S. pilosum* cells are larger than *S. minutum* cells (∼10 μm vs. ∼8 μm in diameter, respectively), and it follows that they would have greater absolute abundances of glycans. However, the relative proportions of different glycans are mostly the same across species.

Despite the similar glycan profiles revealed by the lectin array, we were able to identify a subset of candidate lectins with small but marginally significant fold-change differences in binding among the two tested algal species for further analysis. EEL targets D-galactosyl groups [specifically galactosyl (α-1,3) galactose], and bound 1.07-fold more to symbiotic *S. minutum* than to *S. pilosum* (unadjusted *p* = 0.10). UDA targets high-mannoside N-glycans with a GlcNAc core (Itakura et al., 2017), and bound 1.14-fold more to nonsymbiotic *S. pilosum* than to *S. minutum* (unadjusted *p* = 0.02). Con A (*Canavalia ensiformis* Concanavalin A) targets α-D-mannosyl and α-D-glucosyl groups, and though it did not bind differently to different species, its effect as a general symbiont colonization reducer is well documented in coral-*Symbiodinium* associations (Wood-Charlson et al., 2006; Bay et al., 2011). These three lectins represented the focal molecules for glycan masking during subsequent colonization experiments. For further glycan details, see **Supplementary Figure S1**.

While 1.07- and 1.14-fold changes may seem small, subtle differences in glycan profiles can be biologically important. For example, small changes in human glycosylation can be indicative of cancer status in humans (Kailemia et al., 2017). Although two other lectins (DBA and SNA-I) had larger disparities among symbiont species, we chose not to focus on these for several reasons. DBA targets GalNAc and ManNAc (*N*-acetylmannosamine), while SNA-I targets sialic acid. These specificities are new players, whereas EEL and UDA have specificities previously shown to be important in the symbiosis (Wood-Charlson et al., 2006). In addition, SNA-I has nonspecific-binding patterns and therefore would not be a very selective agent, and its sialic acid target, most often associated with human glycol, is rarely found in nature and has not been detected in previous *Symbiodinium* glycobiology studies (Markell and Trench, 1993).

Although glycan profiles were remarkably similar across species, there were large differences in binding specificity among lectins used in the array (**Figure 1**). For example, the fucose-binding AAL, GalNAc-binding DBA, and mannose-binding Con A all showed relatively high fluorescence response to algal glycans on the array, primarily due to their nonspecific glycan-binding profiles. At the same time, other lectins with similar carbohydrate targets were barely detected. Thus, there was no obvious enrichment for a particular glycan class in *Symbiodinium*, at least based on this 40 lectin array. Future work should include more selective lectin probes to detail N-linked glycan types, incorporate exploration of O-linked glycans, quantify glycans directly, and compare *Symbiodinium* to other symbiotic and nonsymbiotic dinoflagellates to see whether glycan similarity is maintained across larger phylogenetic distances.

### Glycan Recovery Rates

**FIGURE 2:**
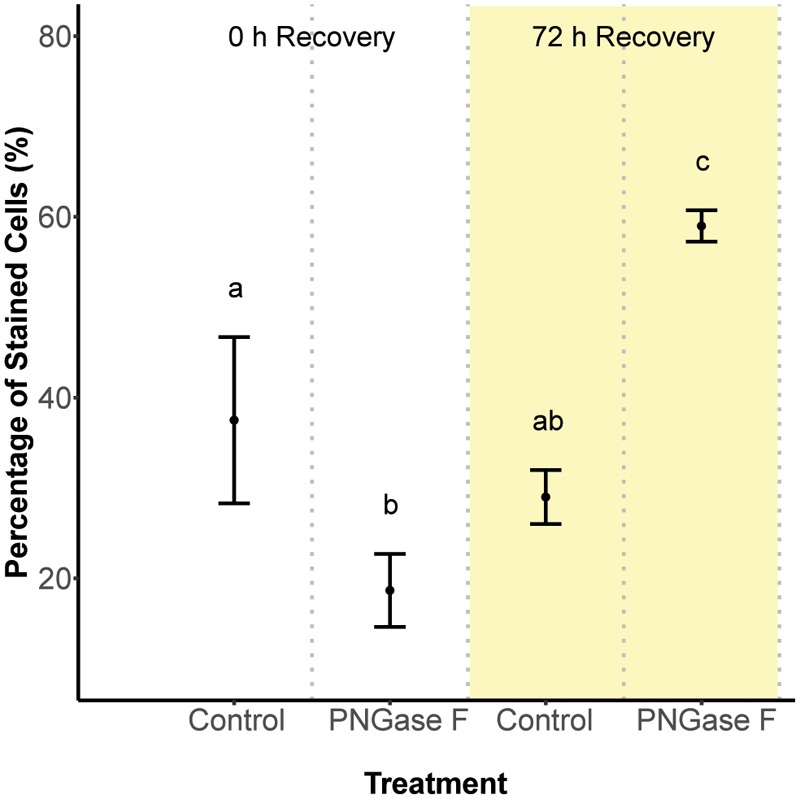
Cell surface glycan recovery time series for *Symbiodinium minutum*. Recovery from cleavage of all N-linked oligosaccharides was measured through binding of the fluorescently tagged lectin Con A either 0 or 72 h after exposure to the amidase PNGase F; controls were exposed to seawater. Letters indicate statistically distinct treatments following ANOVA (*p* < 0.05; *n* = 3 replicates per treatment except for the 0 h control, where *n* = 2). Error bars represent SD.

Next, we hypothesized that glycan turnover at the algal cell surface varied among *Symbiodinium* species, which would affect the time frame over which glycan masking might alter early recognition dynamics between hosts and symbionts. To test this, we used the glycolytic enzyme PNGase F to cleave N-linked glycan residues from the surface of *S. minutum* and *S. pilosum*, then monitored their recovery over time. We exposed the cultures to fluorescently labeled Con A at 0 and 72 h after cleavage, then used flow cytometry to measure fluorescence as a proxy for glycan abundance at each time point. Interestingly, PNGase F did not affect the *S. pilosum* glycome, at least under the conditions we tried (data not shown), meriting future investigation. For *S. minutum*, PNGase F treatment caused glycan abundance to drop initially (**Figure 2**; ANOVA *p* < 0.05). It subsequently recovered, eventually exceeding pre-cleavage levels by 72 h (**Figure 2**; ANOVA *p* < 0.05), perhaps indicating over-stimulation of glycan biosynthesis following artificial removal. An additional experiment using the alternate mannose-binding lectin CVN (*Nostoc ellipsosporum* Cyanovirin N) showed *S. minutum* consistently recovered within 48–72 h (**Supplementary Figure S3**), in line with its doubling time of ∼48 h (Parkinson and Baums, 2014). Although natural turnover rates may differ from this artificial recovery rate, we inferred that the window for effective glycan masking of *S. minutum* was limited to 48 h, and adjusted our experimental approach accordingly. This experiment also showed that under control conditions after 2 h of exposure, Con A masking efficiency (in terms of the percent of cells that were tagged) was 37.5 ± 9.19% SD, while similar experiments showed masking efficiency for EEL was 37.3 ± 1.15% SD, and masking efficiency for UDA was 40.3 ± 5.69% SD.

### Glycan Masking Effects on Colonization

Our primary goal was to determine whether the unique colonization rates of different *Symbiodinium* species could be accounted for by their different glycan profiles, which would suggest a key role of glycan-lectin interactions in mediating symbiosis specificity and establishment. Toward this end, we exposed aposymbiotic host Aiptasia polyps (strain H2) to homologous *S. minutum* cells (strain Mf1.05b) masked with lectins found to bind differentially to the surface of symbiotic and nonsymbiotic *Symbiodinium* species. EEL bound preferentially to symbiotic *S. minutum*, so we predicted *S. minutum* cells masked with this lectin might colonize more slowly than control *S. minutum* cells with no masking (assuming blocking a positive signal should reduce colonization). UDA bound preferentially to nonsymbiotic *S. pilosum*, so while we would still expect *S. minutum* cells masked with this lectin to have a reduced colonization rate, we predicted that the effect would be measurably different than that of EEL. Con A served as potential control that typically reduces *Symbiodinium* colonization rates.

**FIGURE 3:**
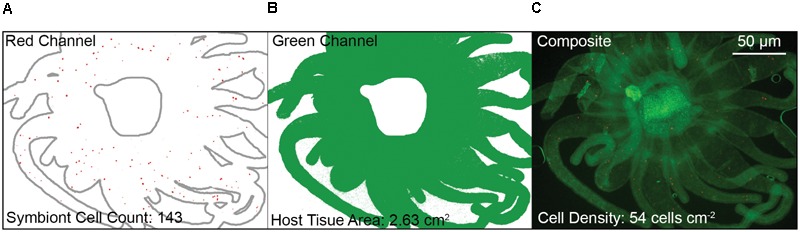
Fluorescence-based quantification of symbiont density in dissected polyp oral disks and tentacles. **(A)** Isolated red channel schematic (*Symbiodinium* chlorophyll autofluorescence) to determine symbiont cell counts, with host outline in gray for clarity. **(B)** Isolated green channel schematic (Aiptasia GFP autofluorescence) to determine host tissue area. **(C)** Composite image of both red and green channels. Note that the polyp mouth area was excluded. Density was calculated as the symbiont cell count divided by the host tissue area.

**FIGURE 4:**
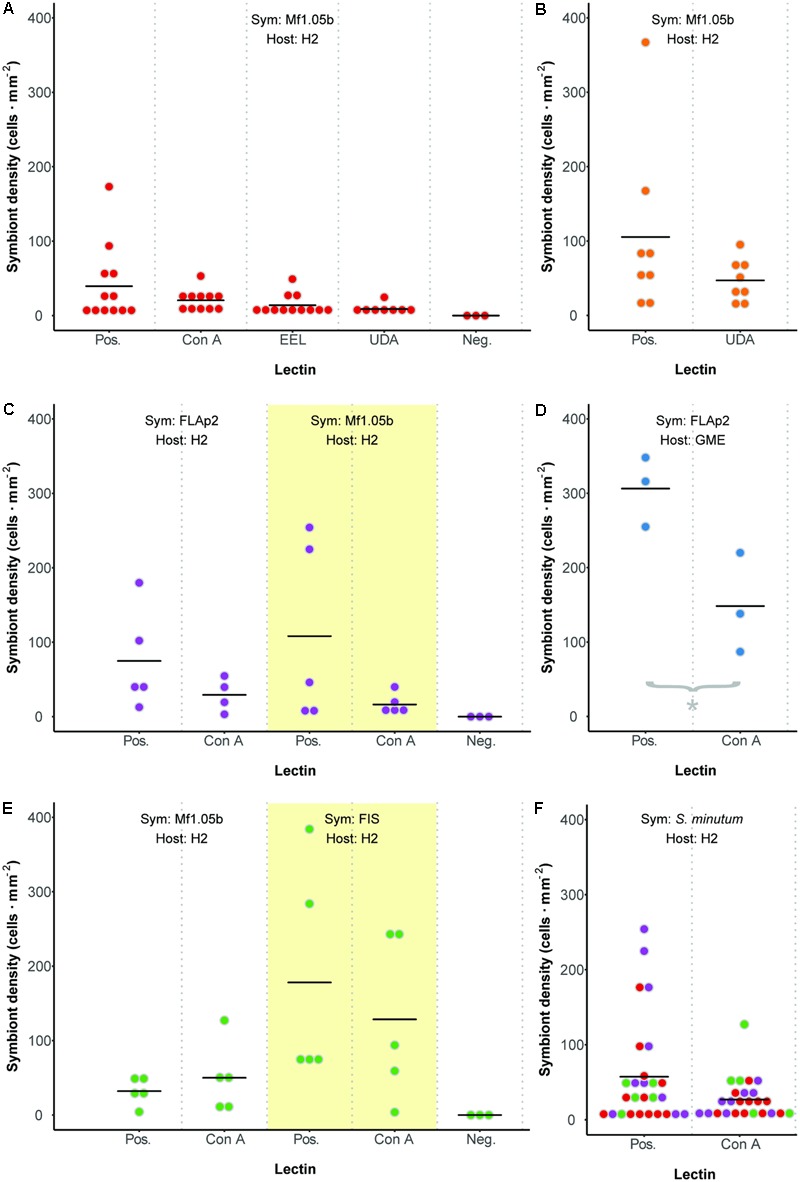
Colonization dynamics: symbiont cell densities in Aiptasia polyps 72 h after initial exposure to *Symbiodinium minutum* treated with various lectins. **(A)** Comparing three lectins of interest. **(B)** An additional trial with lectin UDA and anemones facing prolonged starvation. **(C)** Comparing symbiont strains. **(D)** An additional trial with alternate host strain GME. **(E)** Comparing symbiont cultures and freshly isolated symbionts (FIS) from host H2. **(F)** Aggregate analysis of all comparable data from Pos. or Con A treatments in experiments involving host H2 and symbiont *S. minutum* (strains FLAp2 and Mf1.05b). Dot colors correspond to values from experiments presented in panels **(A)**, **(C)**, and **(E)**. For all experiments, data are grouped by shared host and symbiont strains (indicated at the top of each graph), corresponding to alternately shaded regions. Dots represent data for replicate polyps, while horizontal lines represent mean values for each treatment. Pos. = positive control (polyps were exposed to untreated symbiont cells). Neg. = negative control (polyps were not exposed to symbiont cells).

We developed a fluorescence-based method to normalize cell counts to host surface area, and measured symbiont cell density in each polyp 72 h after initial exposure (**Figure 3**). In contrast to two previous reports (Lin et al., 2000; Wood-Charlson et al., 2006), but largely in agreement with another (Bay et al., 2011), we found no difference in colonization as a result of glycan masking (one-way ANOVA; *F*_3,39_ = 1.99, *p* = 0.13; **Figure 4A**). Untreated cells trended toward higher maximum cell densities, but only in a few polyps; cell densities in the majority of replicates were low and indistinguishable from masked treatments. EEL-, UDA-, and Con A-masked cells trended toward lower cell densities as expected, though not significantly, and the trend seemed similar regardless of lectin identity.

These results were surprising given that Con A has reduced colonization rates consistently in previous studies. One potential explanation for the lack of any pattern was the relatively short inoculation period of 48 h (the narrow window during which glycan masking persists; see **Figure 2**). This may have artificially reduced signal across all treatments by both limiting the opportunity for symbiont uptake and coinciding with the typical lag period in symbiont population growth in new hosts (e.g., Colley and Trench, 1983; Weis et al., 2001). To boost the number of cells present in host tissue, we repeated the experiment with the two most divergent treatments (the unmasked positive control and UDA) after starving polyps for 5 days rather than 1 day. Symbiont cell densities increased in both treatments, but while the average colonization rate for the unmasked cells trended higher once again, the difference remained insignificant (**Figure 4B**). Therefore, the lack of signal was not an artifact of low cell densities in the first experiment.

### Host and Symbiont Strain Effects on Colonization

Seeking alternative explanations for why glycan masking (especially with lectin Con A) did not reduce symbiont uptake significantly in Aiptasia, we tested whether symbiont strain impacted colonization outcomes. Using one host strain (H2), we compared colonization rates for two *S. minutum* strains (Mf1.05b and FLAp2) under two lectin treatments (unmasked or masked with Con A). Again, unmasked symbiont cells trended toward greater average colonization efficiency, but the difference was not significant (**Figure 4C**). Importantly, the patterns were similar regardless of symbiont strain, suggesting that strain identity within a given *Symbiodinium* species does not greatly affect uptake dynamics.

We then tested for a host strain effect by using one of the same *S. minutum* strains (FLAp2) to colonize an alternate host strain (GME), either with or without Con A masking (**Figure 4D**). When compared to **Figure 4C**, it is clear that GME animals generally reached greater symbiont densities than H2 animals regardless of treatment, variation was lower, and the lectin effect was greater. Con A masking significantly reduced colonization by 52% (*t*-test; *p* = 0.029), in line with the 56% reported by Wood-Charlson et al. (2006) for coral larvae but below the 75% reported by Lin et al. (2000) for aposymbiotic Aiptasia polyps. Thus, host strain appears to be an important factor in determining colonization rates in general, perhaps due to unique hosts possessing different numbers of high-mannoside-specific receptors. These results highlight the important contribution of host genetics to symbiosis-related phenotypes, though symbiont genetics also play a major role (Baird et al., 2009; Parkinson and Baums, 2014; Parkinson et al., 2015; Hawkins et al., 2016). Future work should explore host receptor profiling via glycan labeling, pull-down experiments, and host genome mining.

### Host Contamination Effects on Colonization

We noted that most previous tests of cnidarian-algal glycan masking made use of symbiont cells freshly isolated from hosts, rather than cultured cells. FIS typically retain fragments of host material on their outer surface, leading to more dramatic uptake rates (Weis et al., 2001). For example, phagocytosis of FIS by the jellyfish *Cassiopeia xamachana* is one to two orders of magnitude greater than phagocytosis of cultured algae, even if the FIS cells are first heat-killed (Trench et al., 1981; Colley and Trench, 1983). Such results indicate that contaminating host membrane could be a major determinant of early colonization dynamics, and glycan masking in other studies may have reduced uptake by blocking surface molecules primarily on this surrounding host tissue rather than on the algal cells.

We tested for a host contaminant effect by colonizing host polyps (strain H2) with cultured *S. minutum* cells (strain Mf1.05b) or freshly isolated cells (from homogenized H2 animals symbiotic with an uncharacterized *S. minutum* strain), either with or without Con A masking (**Figure 4E**). Although once again we found no significant differences among treatments, FIS treatments trended toward higher cell densities relative to their culture treatment counterparts, but the average reduction due to glycan masking in the FIS treatment was no greater than in our previous H2-Mf1.05b experiments (e.g., **Figures 4A,C**). Thus, while the presence of host tissue on the surface of algal cells does increase uptake overall, it does not appear to alter glycan masking effects, at least within a single host species.

Bay et al. (2011) showed that *S. goreaui* (ITS2 type C1) and an alternate clade D *Symbiodinium* both colonized aposymbiotic larvae of *Acropora tenuis* at different rates and responded to glycan masking somewhat differently. Con A (mannose-binding) and WGA (GlcNAc-binding) had no effect relative to controls in either species, while LPA (Neu5Ac-binding) only affected *S. goreaui*. However, both symbiont species were isolated from different hosts, and while efforts were made to minimize host contamination, it can be difficult to remove host material – particularly symbiosome membranes – without harsh treatment. It is possible that some of the differences among symbiont species could be attributed to their isolation from different hosts, highlighting an advantage of using cultured cells, although such a host effect is not always apparent (Rodriguez-Lanetty et al., 2006). At the same time, cultures are less representative of nature, where coral larvae may be more likely to encounter *Symbiodinium* cells that have been ejected by other hosts in mucus or intact gastrodermal cells as part of the normal process of symbiont population maintenance (Stimson and Kinzie, 1991) or as part of increased post-spawning expulsion (Schwarz et al., 1999, 2002). Such cells likely retain traces of host membrane. Future work should explore the role of host–host glycan-lectin interactions in this context.

### Sample Size Effects

All but one of our colonization experiments yielded no significant differences between controls and treatments. Bayes factor analysis provides a way to assess whether a nonsignificant result counts against a theory (when 0 ≤*B* < 0.3) or merely reflects data insensitivity (when 0.3 ≤*B <* 3) (Dienes, 2014). For our *a priori* prediction that any treatment would differ from at least one other (including controls), the Bayes factor ranged from 0.44 ± 0.01% ≤*B* ≤ 0.88 ± 0.01% depending on the experiment, suggesting insensitivity. In an effort to increase statistical power, we pooled all directly comparable data from Con A or unmasked positive control treatments in experiments involving host H2 and symbiont *S. minutum* (excluding FIS), resulting in a combined 25 and 27 polyps per treatment, respectively (**Figure 4F**). Despite doubling the sample size for the contrast of interest, there remained no statistically significant difference (*t*-test, *p* = 0.17), and no meaningful change in the Bayes factor (*B* = 0.62 ± 0.01%). Thus, there is little reason to believe that increased replication would have improved our ability to detect a difference; the effect size was simply too small or variable given this experimental design.

### Limitations of the Sea Anemone Model and Glycan Masking

Our results highlight some major limitations of using the Aiptasia system as a model for early uptake dynamics with glycan-masked symbionts. The most direct comparison we can make to previous efforts is to the study of Lin et al. (2000), who also used experimentally bleached Aiptasia. Contrary to their work, we found no difference between the uptake of glycan masked and unmasked *Symbiodinium*, leading us to further explore factors that could account for the discrepancy. Some issues were likely methodological. For example, Lin et al. (2000) counted the total number of symbiont cells in the longest three tentacles per sampling point, whereas we imaged the entire oral disk and made efforts to normalize to surface area. They also used polyps collected from the wild, with unknown genetic identity and diversity. Our results showed that some host strains are more readily colonized than others, which is an important consideration for future cnidarian-algal colonization experiments. Importantly, one of the most commonly used strains for Aiptasia *– Symbiodinium* study (H2) turned out to be relatively difficult to inoculate, perhaps owing to its several years of clonal propagation in the laboratory.

More generally, experimentally bleached adult Aiptasia polyps are a very simplified substitute for naturally aposymbiotic larvae when it comes to elucidating the dynamics of initial contact and recognition. Larvae may not yet have developed certain immune components, or their “preferences” may differ from adults. Ontogenetic shifts in symbiont composition are common in cnidarians (Abrego et al., 2009; Poland et al., 2013; Mellas et al., 2014; Poland and Coffroth, 2017), and it is likely that cellular recognition dynamics also shift with age. Bleached adults are also highly variable with respect to re-colonization; even when offered normal, unmasked symbionts, some H2 polyps reached symbiont densities in excess of 200 cells⋅cm^-2^ after 3 days, while others barely exceeded 3 cells⋅cm^-2^. Although we controlled for polyp size, even small size differences may contribute to this variation. Larvae make much better experimental units because they are more similarly sized and so abundant that hundreds or thousands can be assayed at a time, providing more statistical power.

Finally, glycan masking is itself problematic. Our results indicate that cell surface glycan turnover in *Symbiodinium* is rapid. Masking effects may only last for one or two days, so the experimental window for using masked symbionts is narrow. This is not a problem for larvae, which acquire symbionts rapidly and can be assayed early, but adult Aiptasia polyps are slow to recolonize, requiring at least a few days for a reliably high number of symbionts to be detected in the tentacles, and as time passes it becomes harder to link any differences in cell densities to the masking effect. For all of these reasons, we recommend that in the future researchers focus primarily on cnidarian larvae when investigating glycan masking. Alternate approaches to glycan manipulation should also be considered, such as blocking *Symbiodinium* glycan biosynthesis pathways, which may produce a more prolonged effect on the symbiont glycome and facilitate study with adult Aiptasia polyps. In addition, more effort could be placed on investigating the alternative hypothesis that exuded glycoproteins are primarily responsible for establishing symbiosis specificity (Markell et al., 1992; Markell and Trench, 1993; Markell and Wood-Charlson, 2010).

## Conclusion

Glycan-lectin interactions clearly play an important role in early cnidarian host–symbiont dynamics (Lin et al., 2000; Wood-Charlson et al., 2006; Jimbo et al., 2010; Logan et al., 2010; Bay et al., 2011; Davy et al., 2012; Fransolet et al., 2012; Kuniya et al., 2015; Takeuchi et al., 2017). However, glycan masking is not always effective at reducing colonization rates (Bay et al., 2011; this study), and even heat-killed cells, heterologous and/or nonsymbiotic *Symbiodinium* species, and plastic beads can be readily taken up by aposymbiotic hosts, though they do not persist (Trench et al., 1981; Colley and Trench, 1983; Rodriguez-Lanetty et al., 2006; Wolfowicz et al., 2016). In addition, glycan profiles from two very distinct *Symbiodinium* species can be highly similar to each other. These observations are consistent with the idea that glycan-lectin interactions, while likely important for post-phagocytic maintenance of the symbiosis and subsequent winnowing toward specific associations (e.g., Trench, 1988; Weis et al., 2001; Nyholm and McFall-Ngai, 2004; Rodriguez-Lanetty et al., 2006), are not necessarily prescriptive during first contact between cnidarian and *Symbiodinium* cells.

## Ethics Statement

This study was performed on marine invertebrates exempt from ethical review.

## Author Contributions

JP, SL, and VW conceived and designed the experiments. JP, TT, PM, and DA performed the experiments. PM, DA, and SL provided the reagents. JP and TT analyzed the data. JP wrote the paper. JP and PM prepared figures and tables. All authors revised drafts of the paper.

## Conflict of Interest Statement

The authors declare that the research was conducted in the absence of any commercial or financial relationships that could be construed as a potential conflict of interest.

## Footnotes

1 http://www.functionalglycomics.org

## Supplementary Material

FIGURE S1 Lectin specificity of **(A)** Con A, **(B)** EEL, and **(C)** UDA as determined by glycan microarray experiments. Data provided by the Consortium of Function Glycomics. Lectins were labeled with Alexafluor 488 and fluorescence response measured from 611 glycans immobilized on the array.

FIGURE S2 Representative raw images of lectin microarray fluorescence for **(A)** *S. minutum* and **(B)** *S. pilosum*. **(C)** A map of the lectin targets provided by RayBiotech.

FIGURE S3 Cell surface glycan recovery time series for *Symbiodinium minutum*. Recovery from cleavage of all N-linked oligosaccharides was measured through binding of the fluorescently tagged lectin CVN daily up to 96 h after exposure to the amidase PNGase F. Letters indicate statistically distinct treatments following ANOVA (*p* < 0.05; *n* = 3 replicates per treatment except for the 48 h treatment, where *n* = 2). Error bars represent SD. CVN is similar to Con A in that it also target N-linked high-mannose glycans, but CVN has lower nonspecific binding.

TABLE S1 Statistical output for the lectin array from single-channel analysis in the R package ‘LIMMA.’ The log(Fold Change) represents fluorescence intensity in *S. pilosum* (culture ‘Zs’) relative to *S. minutum* (culture ‘Mf 1.05b’). Significant differences (unadjusted *p* ≤ 0.1) are indicated in bold.
