# Supplementary figures and images for: Subtle Differences in Symbiont Cell Surface Glycan Profiles Do Not Explain Species-Specific Colonization Rates in a Model Cnidarian-Algal Symbiosis
Source: Front Microbiol. 2018 May 1;9:842. doi: 10.3389/fmicb.2018.00842 (PMC5938612; doi:10.3389/fmicb.2018.00842)

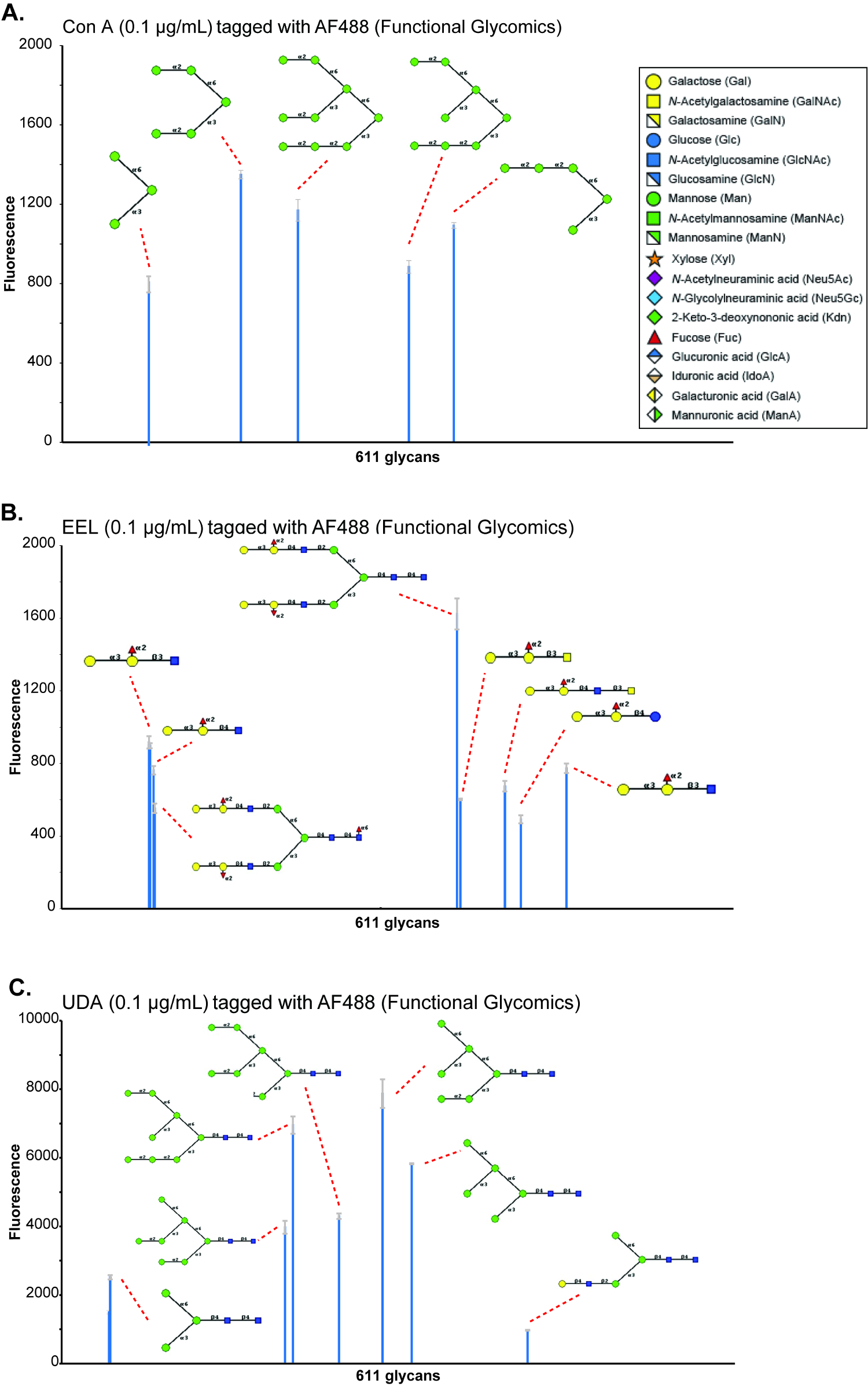

Supplement: FIGURE S1 — Lectin specificity of (A) Con A, (B) EEL, and (C) UDA as determined by glycan microarray experiments. Data provided by the Consortium of Function Glycomics. Lectins were labeled with Alexafluor 488 and fluorescence response measured from 611 glycans immobilized on the array. [file Image_1.TIF]

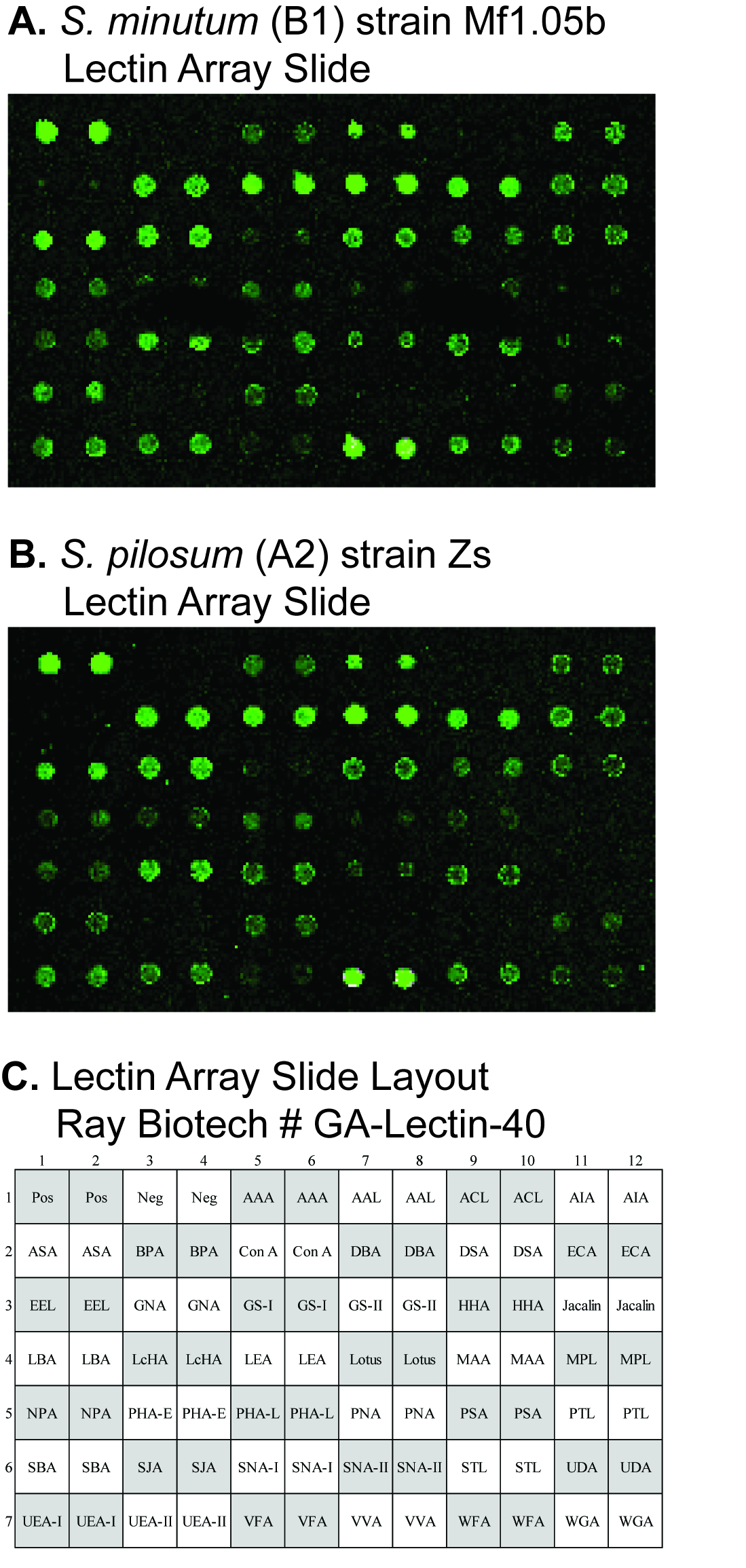

Supplement: FIGURE S2 — Representative raw images of lectin microarray fluorescence for (A) S. minutum and (B) S. pilosum. (C) A map of the lectin targets provided by RayBiotech. [file Image_2.TIF]

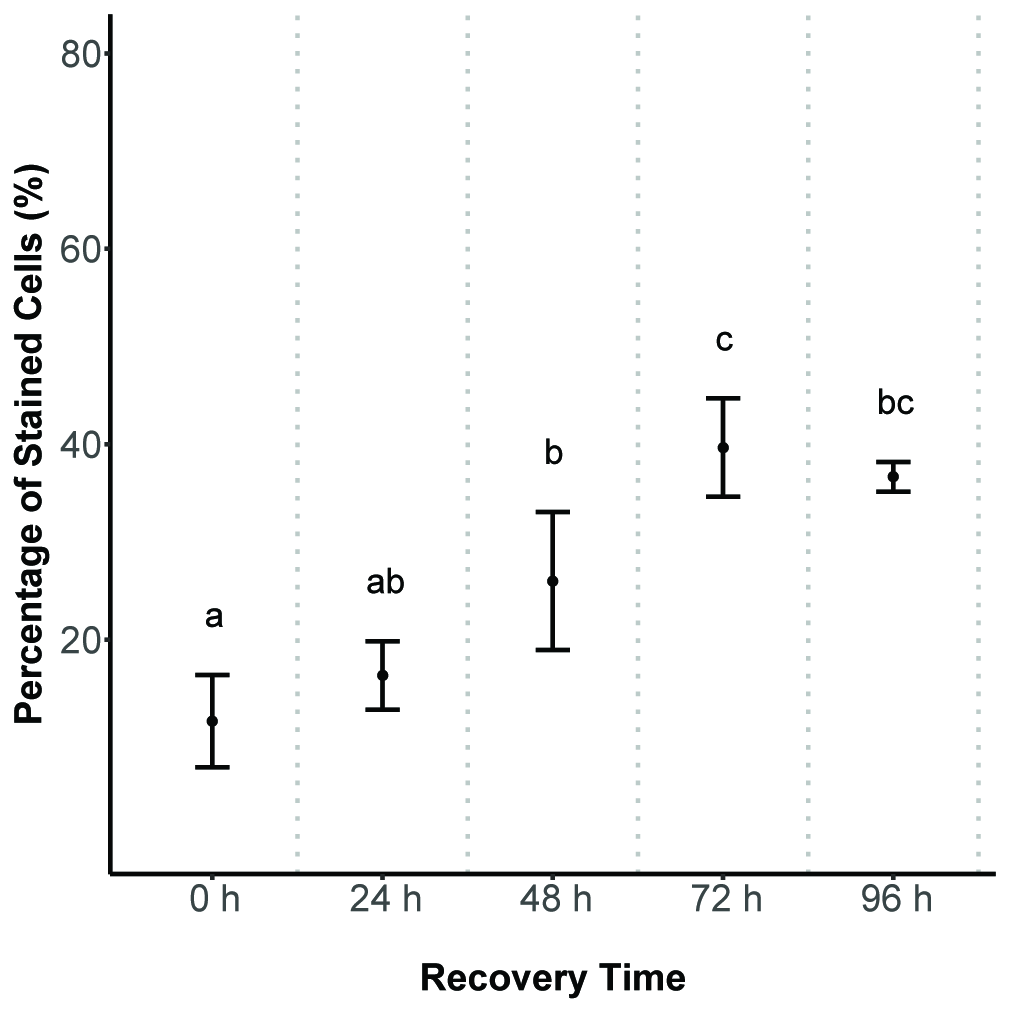

Supplement: FIGURE S3 — Cell surface glycan recovery time series for Symbiodinium minutum. Recovery from cleavage of all N-linked oligosaccharides was measured through binding of the fluorescently tagged lectin CVN daily up to 96 h after exposure to the amidase PNGase F. Letters indicate statistically distinct treatments following ANOVA (p < 0.05; n = 3 replicates per treatment except for the 48 h treatment, where n = 2). Error bars represent SD. CVN is similar to Con A in that it also target N-linked high-mannose glycans, but CVN has lower nonspecific binding. [file Image_3.TIF]
