# Supplementary material for: Subtle Differences in Symbiont Cell Surface Glycan Profiles Do Not Explain Species-Specific Colonization Rates in a Model Cnidarian-Algal Symbiosis
Source: Front Microbiol. 2018 May 1;9:842. doi: 10.3389/fmicb.2018.00842 (PMC5938612; doi:10.3389/fmicb.2018.00842)
Supplement: TABLE S1 — Statistical output for the lectin array from single-channel analysis in the R package ‘LIMMA.’ The log(Fold Change) represents fluorescence intensity in S. pilosum (culture ‘Zs’) relative to S. minutum (culture ‘Mf 1.05b’). Significant differences (unadjusted p ≤ 0.1) are indicated in bold. [file Table_1.docx]

**Supplementary Table 1** Statistical output for the lectin array from single-channel analysis in the R package ‘*LIMMA.*’ The log(Fold Change) represents fluorescence intensity in *S. pilosum* (culture ‘Zs’) relative to *S. minutum* (culture ‘Mf 1.05b’). Significant differences (unadjusted *p* ≤ 0.1) are indicated in bold.

| **Lectin** | **log(Fold Change)** | ***p*-Value** | **Adjusted *p*-Value** |
| --- | --- | --- | --- |
| AAA | -0.05 | 0.49 | 0.81 |
| AAL | 0.46 | 0.24 | 0.81 |
| ACL | -0.20 | 0.54 | 0.81 |
| AIA | -0.22 | 0.14 | 0.81 |
| ASA | -0.06 | 0.57 | 0.81 |
| BPA | -0.07 | 0.46 | 0.81 |
| Con A | -0.09 | 0.44 | 0.81 |
| **DBA** | -0.15 | 0.01 | 0.21 |
| DSA | -0.03 | 0.61 | 0.81 |
| ECA | 0.06 | 0.58 | 0.81 |
| **EEL** | 0.10 | 0.10 | 0.81 |
| GNA | 0.10 | 0.43 | 0.81 |
| GS-I | 0.05 | 0.55 | 0.81 |
| GS-II | 0.10 | 0.21 | 0.81 |
| HHA | 0.00 | 0.99 | 0.99 |
| Jacalin | -0.10 | 0.44 | 0.81 |
| LBA | 0.09 | 0.24 | 0.81 |
| LcH A | 0.00 | 0.99 | 0.99 |
| LEA | -0.03 | 0.67 | 0.82 |
| Lotus | -0.09 | 0.22 | 0.81 |
| MAA | -0.18 | 0.33 | 0.81 |
| MPL | 0.08 | 0.45 | 0.81 |
| NPA | 0.00 | 0.97 | 0.99 |
| PHA-E | 0.20 | 0.37 | 0.81 |
| PHA-L | -0.08 | 0.62 | 0.81 |
| PNA | 0.06 | 0.43 | 0.81 |
| PSA | -0.01 | 0.87 | 0.98 |
| PTL | 0.31 | 0.11 | 0.81 |
| SBA | 0.15 | 0.23 | 0.81 |
| SJA | 0.00 | 0.99 | 0.99 |
| **SNA-I** | -0.17 | 0.02 | 0.21 |
| SNA-II | -0.05 | 0.36 | 0.81 |
| STL | -0.05 | 0.41 | 0.81 |
| **UDA** | -0.19 | 0.02 | 0.21 |
| UEA-I | 0.05 | 0.61 | 0.81 |
| UEA-II | 0.04 | 0.64 | 0.81 |
| VFA | -0.06 | 0.42 | 0.81 |
| WFA | 0.01 | 0.78 | 0.92 |
| WGA | -0.01 | 0.88 | 0.98 |
